# Locust Inspired Algorithm for Cloudlet Scheduling in Cloud Computing Environments

**DOI:** 10.3390/s21217308

**Published:** 2021-11-03

**Authors:** Mohammed Alaa Ala’anzy, Mohamed Othman, Zurina Mohd Hanapi, Mohamed A. Alrshah

**Affiliations:** 1 Department of Communication Technology and Networks, Universiti Putra Malaysia, Serdang 43400, Malaysia; zurinamh@upm.edu.my (Z.M.H.); mohamed.asnd@gmail.com (M.A.A.); 2 Laboratory of Computational Science and Mathematical Physics, Institute for Mathematical Research (INSPEM), Universiti Putra Malaysia, Serdang 43400, Malaysia

**Keywords:** cloud computing, cloudlet scheduling, task allocation, bio-inspired, makespan, resource utilisation, waiting time

## Abstract

Cloud computing is an emerging paradigm that offers flexible and seamless services for users based on their needs, including user budget savings. However, the involvement of a vast number of cloud users has made the scheduling of users’ tasks (i.e., cloudlets) a challenging issue in selecting suitable data centres, servers (hosts), and virtual machines (VMs). Cloudlet scheduling is an NP-complete problem that can be solved using various meta-heuristic algorithms, which are quite popular due to their effectiveness. Massive user tasks and rapid growth in cloud resources have become increasingly complex challenges; therefore, an efficient algorithm is necessary for allocating cloudlets efficiently to attain better execution times, resource utilisation, and waiting times. This paper proposes a cloudlet scheduling, locust inspired algorithm to reduce the average makespan and waiting time and to boost VM and server utilisation. The CloudSim toolkit was used to evaluate our algorithm’s efficiency, and the obtained results revealed that our algorithm outperforms other state-of-the-art nature-inspired algorithms, improving the average makespan, waiting time, and resource utilisation.

## 1. Introduction

Cloud computing is an important trend that has been highlighted by users and industries in relation to cost awareness and convenience in providing computing services as these services are run on a pay-as-you-use basis. Cloud services are known for their ease and speed of access, and are considered ubiquitous since the data centres are distributed all over the world. A new framework has been concurrently enabled in the emerging cloud computing environment, which shifts the physical location of storage and computation of cloud computing networks to reduce operational and maintenance costs [1,2]. This computing model enables convenient, user-friendly, on-request network access to a diversity of shared-pool computing resources distributed across the world, and those resources can be expeditiously released and quickly provisioned to the end-user with reduced effort and management costs [3]. By using virtualisation technology, the cloud platform enables various types of business to run on it, which facilitates easy to use and cost-effective solutions for internet-enabled businesses [4].

There are various service models in the cloud environment, such as platform as a service (PaaS), infrastructure as a service (IaaS), software as a service (SaaS), and XaaS, where X refers to anything as a service (e.g., security as a service) [5], and the terms of these services should be satisfied according to the service-level agreements (SLAs) for the upcoming tasks of the end-users. Executing these tasks requires deploying them on the VMs of a specific physical machine (PM) (i.e., a server) in the cloud computing environment, where the VMs play a vital role in serving the users’ tasks [6,7,8]. Several VMs might be initiated and terminated in the servers, since the cloud services are allocated on a pay-as-you-use basis. Recently, Amazon has developed the elastic cloud, where all the cloud components such as VMs, PMs, and load balancers are expanded on overload and shrink when a machine is under-loaded (i.e., based on the incoming task loads) [9,10]. Therefore, an elastic cloud solution could resolve the resource allocation problems of under-loaded and overloaded VMs/PMs. However, VM efficiency relies on scheduling and load balancing techniques more than the dynamic allocation of resources for execution. Although elasticity enhances cloud performance, these services have boundaries in terms of heterogeneity. For instance, in a heterogeneous cloud environment, cloud resource scaling with different system configurations must be considered. If the cloud necessitates performing work seamlessly, then perfect resource allocation or load balancing should play a vital role in fulfilling the above-mentioned services offered by any cloud computing provider [11,12]. In this regard, nature-inspired techniques have had an impressive impact on allocating resources.

In this work, we have focused on proposing a nature-inspired algorithm. Our algorithm is inspired from the locust to solve a challenging problem of resource allocation and task scheduling. Presently, scholars are paying more attention to nature-inspired algorithms such as genetic algorithms (GAs), ant colony optimisation (ACO), practical swarm optimisation (PSO), and the Bee Algorithm (BeeA). Such nature algorithms incorporate the unique characteristics of creatures in the world that have been keenly observed by researchers and modelled as biologically inspired algorithms [6,9] applicable to engineering problems. Studies by [13,14,15] revealed that better results could be obtained from nature-inspired algorithms than from conventional algorithms.

The main contributions of the proposed methodology lie in attaining the following:

- The methodology provides for a more complete presentation of locust optimisation procedures.
- The design of the discrete version of the locust optimisation algorithm can be implemented for user task scheduling in cloud computing environments.
- Load balancing with efficient task allocation is achieved based on this locust optimisation algorithm, which can efficiently allocate and balance tasks on VMs.
- Allocating resources dynamically by utilising a novel hybrid algorithm using a meta-heuristic (locust-inspired) algorithm allows for efficient scheduling of cloud resources to serve the users’ tasks.
- Evaluation of the proposed method can be performed using resource utilisation, makespan, and waiting time among VMs as performance metrics.

In this paper, our main objective in the development of the proposed task-scheduling locust algorithm is that the tasks should be allocated on the VMs in order to minimise the makespan and waiting time and maximise the resource utilisation. Thus, we mainly concentrate on allocating the users’ tasks using the locust-inspired algorithm while, at the same time, managing the cloud resources. The locust algorithm has been applied to the energy management of cloud computing, as presented by [16,17,18], whereas, to the best of our knowledge, our paper is the first to apply the locust algorithm in the task scheduling field of cloud computing. Our locust algorithm is more efficient compared to other bio-inspired algorithms and therefore outperforms those algorithms in terms of average makespan, waiting time, and resource utilisation.

The remainder of this paper is organised as follows: Section 2 presents an overview of related work. Section 3 presents the proposed algorithm methodology and algorithm modelling. Section 4 describes the simulation tool, simulation configuration, and presents the obtained results with a discussion on the performance evaluation. Finally, we outline the conclusion of our work and the scope of future development in Section 5.

## 2. Related Work

This section analyses and summarises the state-of-the-art task scheduling/allocation algorithms in cloud computing environments using bio-inspired algorithms. Many researchers have been concentrating on bio-inspired algorithms in relation to task scheduling issues, which gets increasingly more attention due to the increasing number of cloud users.

Some researchers have used bee optimisation to enhance the task scheduling approach. Ref. [19] proposed an independent task allocation algorithm for use in multiprocessor system environments named bee colony optimisation (BCO), which was inspired from honeybee foraging. They succeeded by remarkably improving the makespan metric, outperforming the existing approaches. Ref. [20] have improved the load balancing and makespan of cloud computing using a honeybee galvanising algorithm.

Additionally, PSO has been used by some researchers such as [21], who proposed a mathematical model of load balancing and task scheduling (mutation) using PSO, taking into consideration the performance metrics as constraints (makespan, transmission cost, load balancing, reliability, execution time, and round-trip time). The mathematical models in the algorithm proposed by [21] consist of three models. The first model is for calculating the expected execution time (EET) of each task. The second model is for calculating the expected transmission time (ETT) of each task. The third model is for calculating the expected round trip time (ERTT) based on the previous models (EET and ETT). The PSO algorithm has been implemented so that in each iteration, the particle velocity is updated using ERTT (i.e., the third model). However, the authors considered the makespan, while they ignored measurement of resource utilisation, which is another important metric. Finally, a hybrid PSO with a hill-climbing algorithm was presented by [22] for task scheduling to improve the algorithm’s handling of makespan.

Other researchers have used ACO in algorithm development. For example, a multi-objective algorithm based on ACO introduced by [23] is used to schedule user tasks onto suitable resources, where the utilisation of the resources is calculated and the user pays based on usage to minimise the cost in addition to increasing the makespan value. Their methods considered two constraints: the user’s budget and the makespan. The ACO algorithm has achieved improvements in the performance metrics of the cost, makespan, resource utilisation, and deadline violation rate. Additionally, ref. [24] proposed multi-type ant colony optimisation (MACO) incorporating multiple ant types to solve complex multiple land use placement problems.

Combination algorithms are implemented to produce a single algorithm that overcomes the drawbacks of the algorithms they are based upon. A combination algorithm developed by [25] merged ACO with PSO into an algorithm named ACOPS for task scheduling. Additionally, they used historical information in predicting the upcoming workload of the cloud system. The computing time is minimised by satisfying the conditions of the new input request of the tasks. The ACOPS algorithm compares the task memory with the server memory, and if the memory is larger than the remaining memory of the server, then the assignment is rejected. The initial process of the ACOPS algorithm is executed based on ACO, where the ACO search module pre-assigns all the ants (tasks), while the ants’ pheromone function is used in calculating disk utilisation, CPU utilisation, and memory. To improve the searching result, the PSO approach is applied by updating the ant pheromone function of the tasks based on PSO. The algorithm has outperformed its competitors, although the authors neglected the makespan. A self-adaptive ACO algorithm (SAACO) presented by [26] uses PSO to make ACO parameters self-adaptive, which enhances the pheromone update and calculation processes to improve the makespan and load balancing.

Ref. [5] introduced a novel hybrid bio-inspired algorithm for resource management and task scheduling aptly named the HYBRID bio-inspired algorithm, which is based on modified PSO (MPSO) and modified cat swarm optimisation (CSO). Since users’ tasks are received at a rapid rate, cloud providers need an algorithm to handle those tasks in an intelligent manner, and the HYBRID bio-inspired algorithm has been proven superior with benchmarking algorithms such as CSO, RR, MPSO, ACO, and the Exact algorithm in terms of response time, utilisation, and reliability. Ref. [27] introduced the technique of order precedence by similarity to ideal solution (TOPSIS) algorithm, which involves a multi-criteria approach to scheduling tasks onto VMs with a PSO algorithm for calculating the relative closeness of the algorithm criteria. TOPSIS consists of two phases. In the first phase, the algorithm will be dependent on selected scheduling criteria to obtain the tasks’ relative closeness, while in the second phase, the PSO algorithm will initiate the calculation of the relative closeness of task criteria, and each task in each VM will have a calculation for execution time, cost, and transmission-time criteria. To solve the multi-objective task scheduling problem, the TOPSIS algorithm uses a weighted sum of cost, execution time, and transmission time as an objective function. TOPSIS-inspired cost efficiency, as presented by [28], is obtained using a weighted sum of communication time, cost, and execution time to identify the optimal resource among available resources.

Another bio-inspired approach is a human-inspired method proposed by [29] to solve the job shop scheduling problem for task scheduling in a multi-cloud environment. They modelled a known approach, brainstorm optimisation (BSO) [30,31], by contributing self-adaptive characteristics to develop a new algorithm named the Self-Adaptive Brainstorm Optimisation (SA-BSO) scheme. The algorithm was evaluated by comparing it with existing algorithms such as differential evolution (DE), BSO, PSO, and GA, which proved the SA-BSO superior regarding several performance metrics such as makespan, resource utilisation, and job completion rate.

On the other hand, some authors modelled novel nature-inspired algorithms such as the multi-objective, nature-inspired Chaotic Squirrel Search Algorithm (CSSA) proposed by [32] for task scheduling in an IaaS cloud computing environment. The algorithm has been proven superior to benchmarking algorithms in terms of power utilisation, cost, and execution time. Ref. [33] studied crows’ search habits in collecting food, which they attempted to adapt for the cloud computing environment. The manner in which a crow keeps monitoring its mates to discover a better food source is the inspiration for the Crow Search Algorithm’s (CSA) methods of finding suitable VMs for tasks while reducing the execution time of the algorithm. A hybrid meta-heuristic algorithm named FUGE was introduced by [34], which is based on fuzzy theory and a modified version of the standard genetic algorithm (SGA). They have proven the effectiveness of their algorithm mathematically, where they considered VM processing speed, VM bandwidth, memory, and job length for allocating jobs (i.e., sets of tasks) to resources. The performance evaluation is measured based on the execution time, cost, and average degree of imbalance. Ref. [3] proposed a novel meta-heuristic method for profit maximisation of the task scheduling for the private cloud inspired by the bubblenet hunting technique of humpback whales, namely, the Whale Optimisation Algorithm (WOA). Ref. [3] could attain minimum processing time, minimum resource utilisation, high efficiency, and maximum profit of the hybrid cloud to boost the private cloud profit while maintaining the bounded delay-tolerance of the tasks. The experimental result showed that WOA could increase efficiency and maximise profit when compared with ABC and GA algorithms. Ref. [35] introduced a meta-heuristic algorithm, namely, discrete symbiotic organism search (DSOS), to solve numerical optimisation problems. Symbiotic organism search (SOS) mimics the symbiotic relationships (i.e., mutualism, parasitism, and commensalism) of organisms in an ecosystem. New regions are explored in the mutualism phase for better solutions. Parasite vector techniques in the parasitism phase are presented to avoid rapid convergence. They succeeded in enhancing the response time, and makespan and maintained the degree of imbalance in scheduling the tasks on VMs with respect to achieving high resource utilisation with efficient makespan.

Authors’ algorithms have also been inspired from genetics, such as the independent task scheduling based on GA proposed in [36]. Two criteria are considered as a bi-objective minimisation problem in their algorithm: the makespan and energy consumption. They used dynamic voltage scaling (DVS) to reduce the energy consumption, while the unified and double fitness algorithms were proposed for individual selection and to define the fitness function. The main steps of the algorithm are chromosome coding, fitness function calculation, population initialisation, individual selection, mutation operator, and crossover. Hence, they improved the energy consumption and the makespan; however, the degree of imbalance became worse. A hybrid task scheduling algorithm using GA and ACO (GAACO) was proposed [37,38], where [37] considered reliability and time consumption to improve load balancing and makespan. They considered the quality of service (QoS) for the GA as a fitness function. The scheduling process was optimised by combining two bio-inspired algorithms (i.e., GA and ACO). Novel incorporation between GA and ACO for multiple sequence alignment has introduced by [38].

Bacteria algorithms have also been investigated by cloud computing researchers. In [39], a meta-heuristic algorithm for swarm intelligence inspired by bacteria’s foraging and chemotactic behaviour was proposed. The algorithm consists of three main event steps: the chemotaxis event, duplicate event, and elimination-dispersal event. In the chemotaxis step, the two steps used are tumble and move, as those steps are followed periodically during the process of bacterial foraging. Bacterial foraging optimisation could efficiently minimise the VMs’ idle time, energy consumption, degree of imbalance, runtime, and makespan.

Ref. [40] introduced the chaotic social spider algorithm to find the best optimal solution for makespan and utilisation, while [41] proposed a novel task scheduling algorithm inspired by the social spider-mating strategy with respect to QoS awareness. Additionally, the placement of resources was also improved; therefore, they reduced the resource migration with the aim of minimising the cost and execution time while improving other performance matrices such as throughput, reliability, utilisation, turnaround time, execution time, availability, waiting time, execution cost, and convergence when compared with PSO, ABC, and ACO. However, cloudlet parameters were not given consideration, although they are core issues in task scheduling. Moreover, the implementation suffers from cubic time complexities $\left(O\left(n^{3}\right)\right)$.

Refs. [16,17] modelled a server consolidation algorithm based on locust biology, namely, the Locust-Inspired Scheduling Algorithm for Reducing Consumed Energy (LACE). The LACE algorithm is inspired by locust nutrition, regarding which, the algorithm has two phases: solitary and gregarious. The algorithm developed by [16] consolidates VMs on a minimum number of servers, allocates the VMs on a first-come-first-serve basis, and then triggers the migration phase to reallocate the servers that are causing wastage in resources and high execution times; due to the massive number of VM migrations. Ref. [17] improved the CPU utilisation and waiting time. However, the [17] algorithm has the same drawbacks of the LACE algorithm. Ref. [18] presented great improvements on the previous works of [16,17] that were inspired by locusts. They succeeded in enhancing the algorithm in terms of energy consumption, resource utilisation, and VM migrations; however, the algorithm focuses on consolidating the servers.

In this paper, we focus on the scheduling task on the VMs. Our work improves the scheduling of users’ tasks on VMs to obtain full VM/PM utilisation. Previous works [16,17,18] applied the locust algorithm to maintain the energy consumption of the servers, while ours applies locust attributes to schedule users’ tasks and choose the most fitting VMs to handle the users’ tasks. Table 1 illustrates nature-inspired algorithms inspired from different biological phenomena. To the best of our knowledge, no other paper has applied a locust algorithm in task scheduling. Therefore, our paper may be considered the first paper that has applied the locust algorithm on users’ tasks to implement task scheduling inspired by locust behaviour.

## 3. Methodology

### 3.1. Algorithm Modelling

Cloud computing data centres mimic locust swarms in that any data centre contains a large number of heterogeneous VMs which might consist of hundreds or thousands of VMs with different VM specs. Each VM can handle a limited number of heterogeneous tasks, starting from the allocation phase for new task requests. Powerful VMs behave greedily, while weak VMs or unnecessary VMs can serve fewer tasks. A locust algorithm mimics how a locust swarm corresponds to a VM. Locusts consume grass and enervated locusts or grasshoppers when in the swarm phase and mating phase, while a VM allocates tasks from new tasks in the request queue (or from a weak VM when a powerful VM is involved). In the same way as these life-cycle phases of locusts correspond to VM task allocation phases, a scheduling algorithm may be modelled based on locusts’ solitary, mating, and gregarious phases of nutritional consumption. Solitary locusts ordinarily eat grass when they need food, and mating is finding a suitable adult locust, while locusts in a swarm feed gluttonously on grasses, enervated locusts, and grasshoppers. A small swarm might consume more than a ton of crops [42,43].

This feeding behaviour depends on a local search by locusts that are looking for food. Additionally, the mating and gregarious phases are represented as the social interactions of locusts, and the rate of change in the position of a locust can be represented mathematically. We used the following mathematical model to simulate the swarm behaviour of locusts [44]:

(1) $$\dot{x_{i}}=S_{i}+v_{g}+v_{a}$$

There are *N* locusts in the group, which represents the cloudlets, and the *i*th locust has position $x_{i}$. $x_{i}$ as the locust position, the social interactions $S_{i}$, gravity $v_{g}$, and downwind advection $v_{a}$ are each represented in the three phases of the proposed algorithm. The allocation problem of cloudlets can be solved while achieving significant improvements in the makespan, waiting time, and utilisation metrics.

### 3.2. System Model

**Figure 1:**
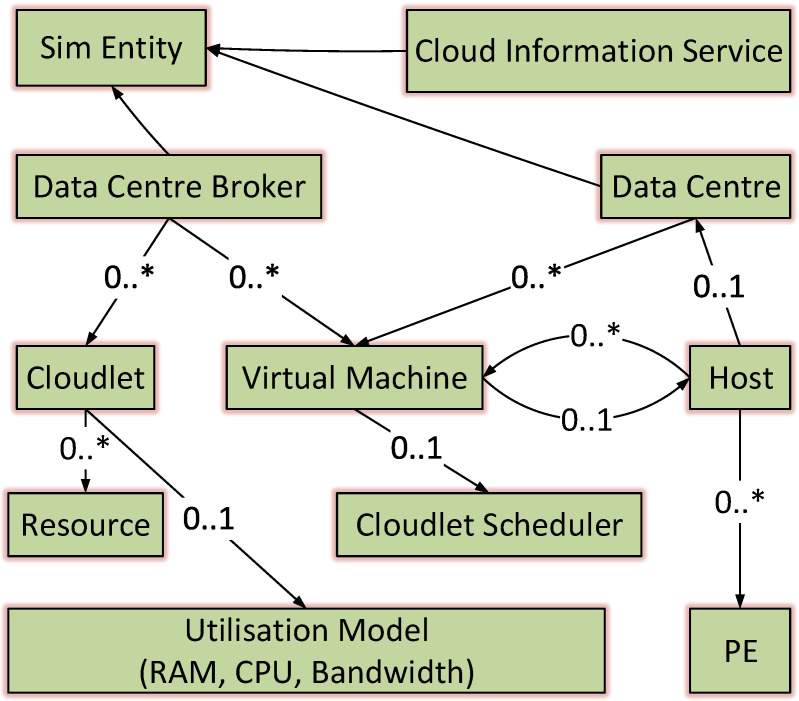
CloudSim component overview.

The extensible modelling of the system is presented in this subsection in addition to the simulation tools of the cloud computing environment. The cloud simulator used in the research was CloudSim, which is a simulation toolkit that provides an excellent framework for both system and behaviour modelling in cloud computing. It evolved from GridSim by GRIDS Laboratory, The University of Melbourne, Australia [45,46]. It is one of the most popular open-source cloud computing simulators and incorporates various Java-based packages. Hence, developers and researchers have proven CloudSim’s efficiency in simulating real cloud computing components in examinations of cloud computing systems. CloudSim toolkit involves numerous components such as its Cloud Information Service (CIS), and components to examine VMs, hosts, data centres, and user-tasks, where the user-tasks are represented in CloudSim as Cloudlets, as depicted in Figure 1. The processes of each VM have their own resources, which are parallel or independent [47]. The components of CloudSim in Figure 1 are summarised as follows:

- Cloud Information Service (CIS): This is the main core entity involved as a registry created by default when the simulation in CloudSim is running. The data centre characteristics are saved in it such as resource availability. The CloudSim broker interacts with it to get resource updates.
- SimEntity: This entity is responsible for handling and sending messages to other entities for each event in the simulation.
- Data Centre (DC): A cloud resource comprising a pool of heterogeneous or homogeneous resources. A DC contains a set of hosts/servers, and its resources are provided to the VMs when required.
- Data Centre Broker: Defined as a VM management handler, the data centre broker acts on behalf of the user as a broker. It handles the processes of creation and destruction of VMs as well as handling task submissions to VMs.
- Physical Machine (PM): This represents a cloud computing server that executes actions related to VM management such as defining policies for bandwidth, memory, and VM processor provisions, and moreover, the creation and destruction processing for VMs.
- Virtual Machine (VM): This is a common means for cloud companies to increase the ability of their servers by running multiple systems on the same physical machine. A VM is given all the system functionalities to execute the end-users’ tasks (cloudlets) through a cloudlet scheduler.
- Processing Element (PE): This acts as a processor unit, which can be {1,2,3, ...}.
- Utilisation Model: This parameter is a determiner of the resource utilisation of the processor (e.g., if it is set to full, then the task will utilise all available resources of the VM, whereas if it is set to stochastic, then a random utilisation will be generated every time span).

**Figure 2:**
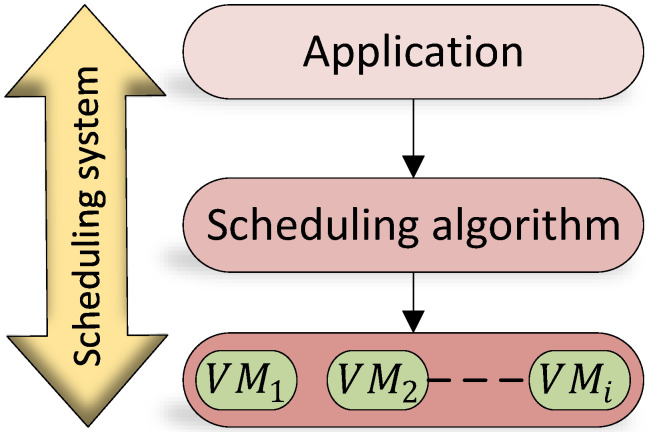
Scheduling system overview.

The scheduling system consists of three modules [48], as illustrated in Figure 2. The cloudlets (i.e., tasks) represent the application in the scheduling system, which is the first module, while the second module is the scheduling algorithm that controls the cloudlet allocation (i.e., how and where the cloudlets should be allocated onto the VMs) by estimating the time required and through other calculations. The third module comprises the VMs that are used to handle the tasks and perform the operations requested by users.

### 3.3. The Proposed Algorithm

**Figure 3:**
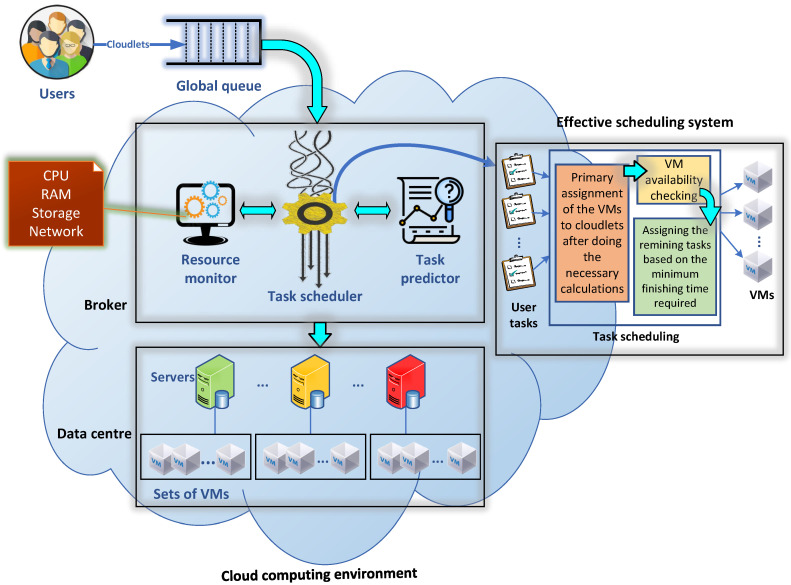
Proposed algorithm architecture.

This paper focuses on the problem of scheduling tasks on virtual machines to minimise the task scheduling execution time. The algorithm efficiency can be presented by measuring the maximum time consumed for each VM to complete the assigned tasks within a given time frame or set of tasks that have been sent to the host, which is called the makespan for that VM or the VM completion time [49]. The of average makespan of all VMs represents the average makespan of a specific host. The proposed algorithm architecture is illustrated in Figure 3.

The system initially receives a set of *m* virtual machines, $VM=\{VM_{1},VM_{2},\ldots ,VM_{m}\}$ that are occupied by set of *n* tasks *T*, where $T=\{T_{1},T_{2},\ldots ,T_{n}\}$, $m,n\in Z^{+}$. The VMs should process *n* tasks.

The virtual machines are parallel and independent, and the scheduler allocates independent tasks to these VMs. Additionally, processing a task on a VM cannot be interrupted (i.e., non-preemption). Reducing the makespan is considered the main objective of the task scheduling for cloud computing. The makespan is the highest completion time in the VM. The completion time is defined as follows:

**Definition** **1.** *Let CT be a set of completion times where $CT=\{CT_{1},CT_{2},\ldots ,CT_{n}\}$, such that each $T_{i}$ on $VM_{j}$ has a different $CT_{i}$, where 1≤i≤n and 1≤j≤m. Hence, the makespan for $VM_{j}$ is defined using Equation (2):*

(2) $$Makespan_{j}=max\left(CT_{i}\right)\;\;|\;\;1\leq i\leq n\;\;and\;\;1\leq j\leq m,$$

*where the average makespan is defined using Equation (3):*

(3) $$AverageMakespan\left(MS\right)=\frac{\sum_{j=1}^{m}Makespan_{j}}{m}.$$

The common source of user dissatisfaction is the waiting time, which should be maintained at the lowest level by the service providers throughout providing enough services acted on by the VMs or servers. Additionally, the service providers must be willing to reimburse the users once the waiting time has exceeded a certain limit. The waiting time gets increased when the targeted VMs get too busy to handle the cloudlets. The waiting time of $VM_{j}$ is defined using Equation (4):

(4) $$WaitingTime_{j}=\sum_{i=1}^{x}PT_{i},\;\;\;\;\forall \;\;x\in T,$$

where *j* is the number of VMs and $PT_{i}$ is the processing time of $T_{i}$. The average waiting time is defined using Equation (5):

(5) $$Average\;WaitingTime\left(WT\right)=\frac{\sum_{j=1}^{m}WaitingTime_{j}}{m}.$$

The output of the proposed algorithm is the execution time plan, which, when enforced, would result in a minimum average makespan for all VMs. It is one of our vital motivations to reduce the average makespan and average waiting time, while utilisation and load balancing are other factors that will be maintained. Therefore, the objective function of the proposed algorithm is as follows:

(6) $$f=max\left(U+\frac{1}{\left|MS\right|}+\frac{1}{\left|WT\right|}\right),\;\;\forall \;\;U\leq 1.0,$$

where MS is the average makespan and WT is the average waiting time for the execution time plan, *U* is the VM utilisation. *f* is inversely proportional to MS and WT and directly proportional to the VM utilisation. The higher the average of makespan and waiting time, the smaller the value of *f*, while the larger the utilisation (*U*) value is, the larger the value of *f* will be.

Next, we sort the VMs based on their processing speed in case the VMs have different processing speeds. The processing speed in simulation-based is measured in MIPS units denoting a million instructions per second. Our algorithm consists of three phases, which are described in the following subsections in more detail.

#### 3.3.1. Preliminary Selection of VMs

**Figure 4:**
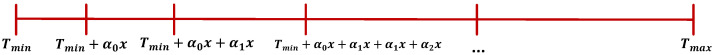
Range distributions of cloudlets among VMs.

The first step in the algorithm is to select an outfit VM for each cloudlet based on cloudlet length. Initially, the data centre broker (DCB) will perform intensive computations to bind a cloudlet with a VM. Hence, to determine a suitable VM, we need to compute the phase parameters as illustrated below:

1 Computing the maximum and minimum cloudlet length ($T_{max}$ and $T_{min}$, respectively).
2 Finding the total processing speed of all VMs represented in MIPS units as in Equation (7).
  (7) $$TotalVMsSpeed=\sum_{j=1}^{m}mips_{j}$$
3 Computing the difference (*x*) between $T_{max}$ and $T_{min}$ over the total of mips as in Equation (8).
  (8) $$x=\frac{\left(T_{max}-T_{min}\right)}{TotalVMsSpeed}$$
4 If the VMs have different processing speeds, they will be sorted in increasing order based on their processing speeds (i.e., $VM\;MIPS_{j+1}>VM\;MIPS_{j}$).
5 We will assume $\alpha_{i}$ is a MIPS of $VM_{j}$ to find the acceptance range, as illustrated in Figure 4.
6 Computing the lower and upper cloudlet-length boundaries for each VM (i.e., each VM has an accepted length range of cloudlets) with a linear sequential equation as per Equation (9) and Equation (10), respectively:
  (9) $$T_{min}+\alpha_{0}x+\alpha_{1}x+\cdots +\alpha_{m-1}x+1$$
  (10) $$T_{min}+\alpha_{0}x+\alpha_{1}x+\cdots +\alpha_{m}x$$
  The acceptance range equations for VMs are illustrated in Table 2.

To this end, each cloudlet will initially be identified according to the targeted VM it is to be served on. When the lower and upper boundaries are set, the DCB will allow the stream of cloudlets to flow into the VMs.

#### 3.3.2. Checking VM Utilisation

In this phase, the DCB will check the assigned VM availability and the total processor utilisation. The checking of the VMs will start from the next following VM if these were unavailable because of high utilisation. Once all VMs that follow the targeted VM are checked, and they are unavailable or highly utilised, then the VMs prior to the targeted one will be checked too. For instance, if the targeted VM was $VM_{j}$, then the DCB will be started from $VM_{j+1}$ to $VM_{j+m}$, and if they are all underutilised, then the DCB starts checking from $VM_{j-1}$ to $VM_{0}$. To this end, if there are cloudlets still unallocated, then the following phase must find suitable VMs for them.

#### 3.3.3. Earlier Cloudlet Handling

This phase can eliminate all unassigned cloudlets by finding the VM that can accomplish its cloudlet processing and the coming cloudlets. For example, #cloudlet10 should be assigned to #VM8, but if #VM8 is busy with processing other cloudlets, then #cloudlet10 will check #VM9, #VM10, ..., #VMm. Once all are busy, then #VM7, #VM6, ...will be checked until finding a free VM to handle that cloudlet. If all are busy, the algorithm will compute the required finishing time of that cloudlet on all VMs while taking into consideration that each VM has a set of cloudlets in their queues that are waiting to be handled. Therefore, the computation of the finishing time will be based on measuring the finishing time for all cloudlets of the specific VM plus the finishing time of #cloudlet10. Equation (11) calculates the current finishing time required for a specific cloudlet ($T_{y}$). After obtaining all the current finishing times of all VMs for #cloudlet10, then the smallest finishing time required will be selected as a handling VM for that cloudlet.

(11) $$FinishingTimeVm_{j}=\sum_{i=1}^{x}PT_{i}+T_{y}\;\;\;|\;\;\;WaitingTime_{j}+T_{y}\;\;\;s.t.\;\;\;1\leq x\leq n\;\;and\;\;1\leq y\leq n.$$

This phase is the most vital phase in the algorithm, where it can maintain the VM loads and make the VMs more flexible to accommodate the cloudlets, where the accommodation of cloudlets will be based on VM processing speed (i.e., high VM processing speed can accommodate a high number of cloudlets). Thus, fair scheduling can result in high VM utilisation in addition to getting the best finishing time for all VMs (i.e., best makespan overall). Algorithm 1 shows the pseudocode for our algorithm.
**Algorithm 1:** Locust scheduling algorithm. **Input**: VM configurations; cloudlet configurations **Output**: Optimised allocation1 **Precondition:** Identify 
$T_{max},\;T_{min},\;TotalMIPS_{VM}\;\left(7\right),\;x\;\left(8\right),\;\alpha ,\;lowerlimits\;\left(9\right)\;\&\;upperlimits\;\left(10\right)$ ;

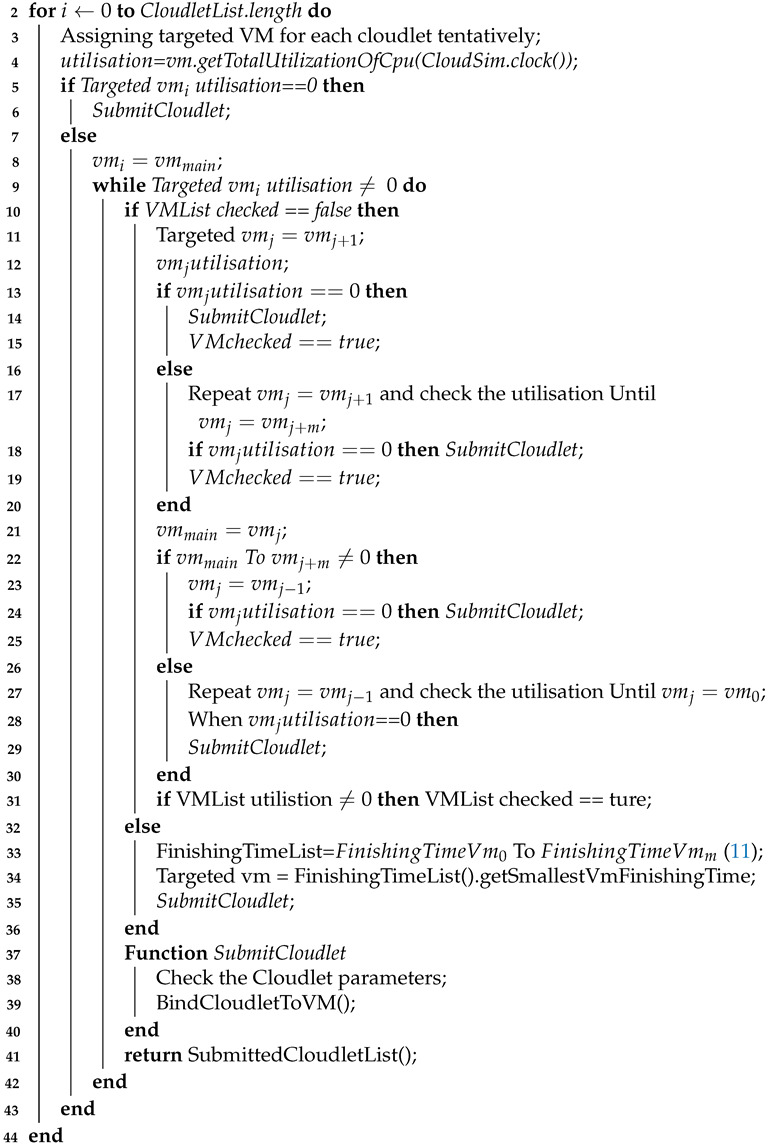



## 4. Experimental Results

In this section, we present the experimental results, the simulation parameters, and the simulation’s data. For the purpose of comparison, 10 separate runs were made to obtain the statistical results.

### 4.1. Simulation Tool

Providing facilities for modelling and simulating network connectivity and resources requires various configurations, capabilities, and domains, which can be provided by the CloudSim toolkit. Additionally, interfaces, information services, and the primitives for application composition are supported for assigning cloudlets to resources. We used the CloudSim toolkit for simulating our algorithm due to the many advantages of it, such as those referred to by [47,50]:

- CloudSim allows the modelling of heterogeneous resources.
- The number of cloudlets that represent user applications is unlimited.
- Many of the cloud computing entities require simultaneous handling.
- CloudSim analysis methods can register all the required operations and calculate the statistics of the selected metrics.
- The simulator supports both static and dynamic schedulers.

Heterogeneity is the main feature of the cloudlet application, which can be represented by I/O intensive or processors.

### 4.2. Simulation Configurations and Parameters

Due to the cost of practical experimentation and the variance in cloud computing environments, the CloudSim Simulator based on the Java programming language was used to validate our algorithm. The proposed algorithm was evaluated and compared with other algorithms using different parameters for PMs, VMs, and cloudlets. The locust parameters are shown in Table 3. We used up to 40 cloudlets in the first experiment in order to calculate metrics such as the average makespan, waiting time, and resource utilisation. In the second experiment, we used up to 500 cloudlets and compared our algorithm with different nature-inspired algorithms regarding the makespan metric. Makespan improvement can prove the system efficiency as it confirms the ability to accomplish the cloudlet processes on time, which can assure users’ satisfaction with the cloud provider service.

### 4.3. Comparison Results

To study the algorithm performance and to validate its efficiency, vital metrics were chosen to evaluate the algorithm performance, such as average makespan, average waiting time, and resources utilisation. Two types of experiments were used to provide the attained results, which are discussed below.

#### 4.3.1. Type 1 Experiment (Locust Inspired Algorithm vs. TOPSIS–PSO)

For the first type of experiment, we compared our approach with the state-of-the-art algorithm TOPSIS-PSO in terms of average makespan, average waiting time, and resource utilisation. The parameter settings are provided in Table 3.

##### Analysis of Makespan

**Figure 5:**
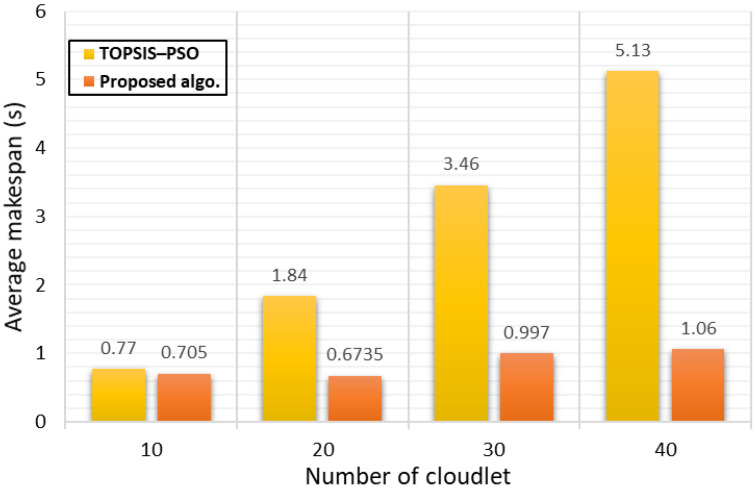
Average makespan.

The makespan metric is defined as the highest completion time of a cloudlet in the VM. In other words, it is the execution time required to process all the cloudlets in a VM queue [8,49]. The behaviour of the proposed algorithm is shown in Figure 5 as compared with a state-of-the-art algorithm TOPSIS–PSO [27]. The TOPSIS–PSO algorithm was inspired by the Practical Swarm Optimisation Algorithm, becoming a replacement for the traditional PSO algorithm. Therefore, we chose TOPSIS–PSO [27] for our comparison. Figure 5 shows an immense improvement in the average makespan of our algorithm, where as much as the cloudlet was increased, the makespan improvement was increased, too, due to the efficient utilisation of the VM resources.

Also, the third phase of the algorithm aided in maintaining the makespan to keep it as low as possible. Since the makespan is related to the overall execution time of the algorithm, getting the lowest makespan is better.

##### Analysis of Waiting Time

**Figure 6:**
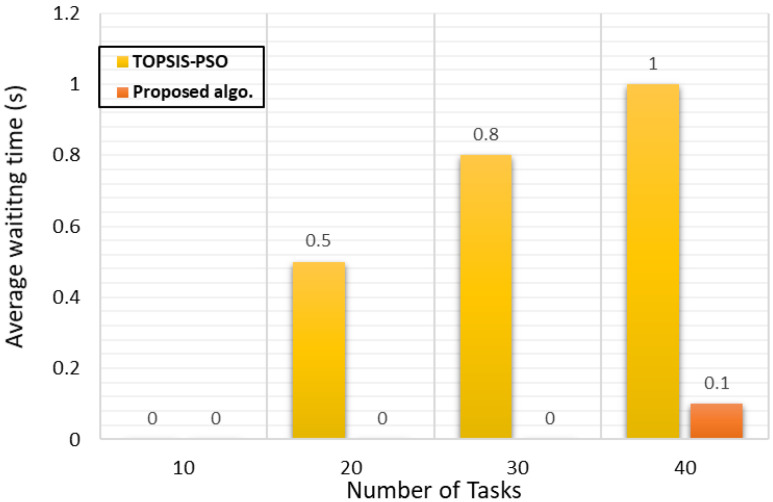
Average waiting time.

The waiting time metric is the period of time that the cloudlet will be waiting until it gets processed. Figure 6 shows the average waiting time of the cloudlets to be submitted to the VMs, where our algorithm overcomes the efficiency of TOPSIS-PSO due to selecting the best VM to process a cloudlet. The algorithm was run in **TimeShareScheculing** type, which means a set of cloudlets was running in parallel, depending on the VM resources capability and scheduling policy. Therefore, when there are a number of cloudlets submitted to a VM, all of them would be running at the same time and the waiting time for them would be equal to 0, especially if the scheduling achieved efficient load balancing for all VMs.

##### Analysis of Resource Utilisation

**Figure 7:**
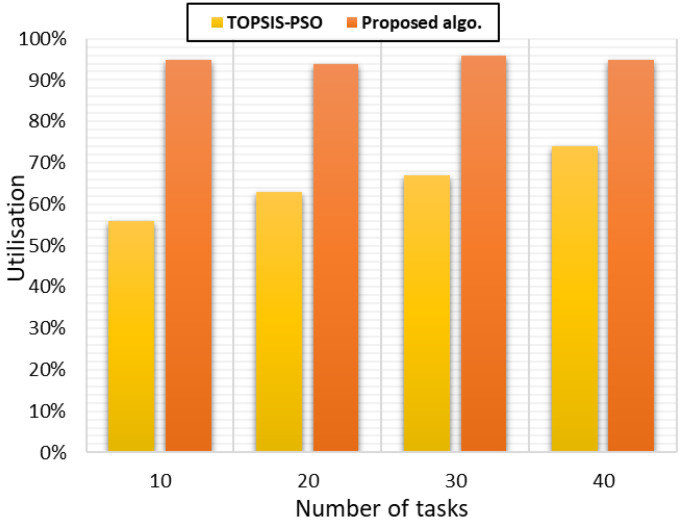
VM utilisation.

The resource utilisation is that which the cloudlets will consume from the VMs. An efficient scheduling algorithm can allocate cloudlets on VMs in a way that assures the perfect placement of each cloudlet on a VM that could result in improving in the system performance. At the same time, the algorithm should assure the highest level of exploitation of the resources. Figure 7 shows the utilisation percentage of the VMs were at the highest levels of VM utilisation. Additionally, the scheduling policy achieved a high level of load balancing leading to efficiently utilising the VM resources, as well.

#### 4.3.2. Type 2 Experiment (Locust Inspired Algorithm vs. State-of-the-Art Algorithms)

In the second experiment, we chose the makespan as a performance evaluation metric since it is considered a vital metric in the cloudlet scheduling area. We used up to 500 cloudlets and compared our algorithm with different nature-inspired algorithms regarding the makespan metric. Makespan improvement can prove system efficiency due to its ability to accomplish the cloudlet processing on time, which can assure user satisfaction with the cloud provider service.

**Figure 8:**
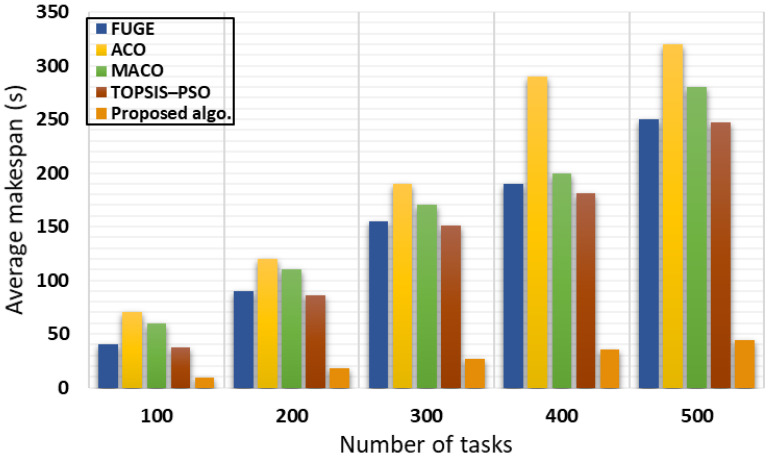
Average makespan.

Our algorithm was compared with different existing and state-of-the-art algorithms such as TOPSIS–PSO [27], FUGE [34], ACO [51], and MACO [52]. Table 4 shows the CloudSim configurations for the second experiment. The obtained results for the average makespan with respect to executed tasks are illustrated Figure 8. The results of the proposed algorithm show an efficiency improvement due to the efficient allocation of the users’ tasks, where all incoming tasks were allocated according to the lowest waiting time for a VM. Therefore, the tasks were executed as quickly as possible.

## 5. Conclusions and Future Work

This article has presented a biological algorithm inspired by locusts for use in resolving cloudlet scheduling problems in the cloud computing environment. The use of a locust algorithm is considered novel in this area (i.e., cloudlet scheduling area). Cloudlet scheduling plays a key role in delivering good service to cloud computing users. The algorithm consists of three phases to efficiently allocate the users’ tasks onto VMs and maintain load balancing of the VMs and PMs. In the first phase, preliminary selection of the VMs is made and then a VM monitoring approach is initiated to keep all the servers under control. Once the VMs are busy with handling the tasks, the earliest finishing times of the tasks, including the times required for the coming tasks, are computed to find the minimum required time needed to assign a cloudlet to a VM. The results obtained by the proposed algorithm were proven more efficient in comparison with other nature-inspired algorithms. We used the makespan as the main performance metric, while in addition, the waiting time and resource utilisation where metrics used to prove the algorithm’s efficiency would achieve user satisfaction. Finally, our results show that cloudlet scheduling using the proposed algorithm was done successfully with the optimisation of resource usage and outperformed other state-of-the-art algorithms.

In the future, a non-linear optimisation technique associated with soft computing will be developed in another version of the proposed algorithm. The use of a learning mechanism will deal with the cloudlet scheduling challenges in an intelligent way by predicting the fluctuations of VM resources that may occur during or after submitting cloudlets to a VM. Additionally, we will present the performance evaluation for different metrics of the locust algorithm.

## Figures and Tables

**Table 1:** Related work summarisation.

Ref.	Year	Algorithm Type	Improvement Types		Metrics		
			TS	SC	MS	WT	U
[19]	2012	BCO	✓		✓		
[20]	2015	ABC	✓		✓		
[21]	2015	PSO	✓		✓	✓	
[22]	2018	PSO & hill-climbing	✓		✓		
[23]	2015	ACO	✓		✓		✓
[24]	2012	MACO	✓		✓		✓
[25]	2015	ACO with PSO (ACOPS)	✓			✓	
[26]	2015	ACO & PSO to generate SA-ACO (self-adaptive)	✓		✓		
[5]	2017	MPSO & CSO	✓			✓	✓
[27]	2019	TOPSIS (PSO based)	✓		✓		✓
[28]	2020	TOPSIS (PSO based)	✓		✓		
[31]	2011	BSO	✓		✓		✓
[30]	2017	BSO	✓		✓		✓
[29]	2020	SA-BSO	✓		✓		✓
[32]	2020	CSSA	✓		✓		
[33]	2020	CSA	✓		✓		
[34]	2015	Fuzzy & SGA (FUGE)	✓		✓		
[3]	2020	Bubble-net hunting of humpback wales (WOA)	✓		✓		✓
[35]	2016	DSOS	✓		✓		✓
[36]	2012	GA	✓		✓		
[38]	2008	GA & ACO	✓		✓		
[37]	2015	GA & ACO	✓		✓		
[39]	2020	Bacteria & chemotactic phenomenon	✓		✓		
[40]	2019	Chaotic social spider	✓		✓		✓
[41]	2020	social spider-mating (SSRPA)	✓		✓	✓	✓
[16]	2018	Locust		✓			✓
[17]	2019	Locust		✓		✓	✓
[18]	2021	Locust		✓			
Our algorithm	2021	Locust	✓		✓	✓	✓

Note: TS refers to task scheduling, SC = server consolidation, MS = makespan, WT = waiting time, U = resource utilisation. The table is sorted/divided based on the algorithm type.

**Table 2:** The acceptability ranges of the cloudlet length.

#VM	Minimum *T* Length	Maximum *T* Length
$VM_{0}$	$T_{min}$	$T_{min}+\alpha_{0}x$
$VM_{1}$	$T_{min}+\alpha_{0}x+1$	$T_{min}+\alpha_{0}x+\alpha_{1}x$
.	.	.
.	.	.
.	.	.
$VM_{m}$	$T_{min}+\alpha_{0}x+\alpha_{1}x+\cdots +\alpha_{m-1}x+1$	$T_{min}+\alpha_{0}x+\alpha_{1}x+\cdots +\alpha_{m}x=T_{max}$

**Table 3:** Simulation parameters.

Parameter	Value
No. of Cloudlets	10–40
Cloudlet length	(100–2500) MI
No. of VMs	10
VM MIPS	2400 MIPS
Task scheduler	Time-shared
No. of hosts	1
Host(s) Storage	1,000,000 MB
Host(s) memory	4096 MB
No. of data centres	1
pesNumber (No. of CPUs)	5
num_user (No. of users)	1
Utilisation model	Full utilisation
System architecture	X86
Operating system	Linux
VMM	Xen

**Table 4:** Simulation parameters for type 2 experiment.

Parameter	Value
Total number of tasks	100–500
Length of tasks	1000–20,000
Total number of VMs	50
VM memory (RAM)	256–2048
VM bandwidth	500–1000
Number of PEs required	1–4
Number of DCs	10
Number of hosts	2–6

## Data Availability

The data presented in this study are available on request from the corresponding authors.
